# The Many Faces of Covid-19 at a Glance: A University Hospital Multidisciplinary Account From Milan, Italy

**DOI:** 10.3389/fpubh.2020.575029

**Published:** 2021-01-08

**Authors:** Alberto Priori, Alessandro Baisi, Giuseppe Banderali, Federico Biglioli, Gaetano Bulfamante, Maria Paola Canevini, Maurizio Cariati, Stefano Carugo, Marco Cattaneo, Amilcare Cerri, Davide Chiumello, Claudio Colosio, Mario Cozzolino, Antonella D'Arminio Monforte, Giovanni Felisati, Daris Ferrari, Orsola Gambini, Marco Gardinali, Anna Maria Marconi, Isotta Olivari, Nicola Vincenzo Orfeo, Enrico Opocher, Luca Pietrogrande, Antonino Previtera, Luca Rossetti, Elena Vegni, Vincenzo Toschi, Massimo Zuin, Stefano Centanni

**Affiliations:** ^1^Neurology, Department of Health Sciences, San Paolo University Hospital, Azienda Socio Sanitaria Territoriale Santi Paolo e Carlo, University of Milan Medical School, Milan, Italy; ^2^Thoracic Surgery, Department of Health Sciences, San Paolo University Hospital, Azienda Socio Sanitaria Territoriale Santi Paolo e Carlo, University of Milan Medical School, Milan, Italy; ^3^Pediatrics, Department of Health Sciences, San Paolo University Hospital, Azienda Socio Sanitaria Territoriale Santi Paolo e Carlo, University of Milan Medical School, Milan, Italy; ^4^Maxillofacial Surgery, Department of Health Sciences, San Paolo University Hospital, Azienda Socio Sanitaria Territoriale Santi Paolo e Carlo, University of Milan Medical School, Milan, Italy; ^5^Pathology, Department of Health Sciences, San Paolo University Hospital, Azienda Socio Sanitaria Territoriale Santi Paolo e Carlo, University of Milan Medical School, Milan, Italy; ^6^Pediatric Neuropsychiatry, Department of Health Sciences, San Paolo University Hospital, Azienda Socio Sanitaria Territoriale Santi Paolo e Carlo, University of Milan Medical School, Milan, Italy; ^7^Radiology Unit, Azienda Socio Sanitaria Territoriale Santi Paolo e Carlo, Milan, Italy; ^8^From the Units of Cardiology, Department of Health Sciences, San Paolo University Hospital, Azienda Socio Sanitaria Territoriale Santi Paolo e Carlo, University of Milan Medical School, Milan, Italy; ^9^Internal Medicine, Department of Health Sciences, San Paolo University Hospital, Azienda Socio Sanitaria Territoriale Santi Paolo e Carlo, University of Milan Medical School, Milan, Italy; ^10^Dermatology, Department of Health Sciences, San Paolo University Hospital, Azienda Socio Sanitaria Territoriale Santi Paolo e Carlo, University of Milan Medical School, Milan, Italy; ^11^Intensive Care, Department of Health Sciences, San Paolo University Hospital, Azienda Socio Sanitaria Territoriale Santi Paolo e Carlo, University of Milan Medical School, Milan, Italy; ^12^Workers' Health, Department of Health Sciences, San Paolo University Hospital, Azienda Socio Sanitaria Territoriale Santi Paolo e Carlo, University of Milan Medical School, Milan, Italy; ^13^Nephrology & Dialysis, Department of Health Sciences, San Paolo University Hospital, Azienda Socio Sanitaria Territoriale Santi Paolo e Carlo, University of Milan Medical School, Milan, Italy; ^14^Infectious Disease, Department of Health Sciences, San Paolo University Hospital, Azienda Socio Sanitaria Territoriale Santi Paolo e Carlo, University of Milan Medical School, Milan, Italy; ^15^Otorhinolaryngology, Department of Health Sciences, San Paolo University Hospital, Azienda Socio Sanitaria Territoriale Santi Paolo e Carlo, University of Milan Medical School, Milan, Italy; ^16^Oncology Unit, Azienda Socio Sanitaria Territoriale Santi Paolo e Carlo, Milan, Italy; ^17^Psychiatry, Department of Health Sciences, San Paolo University Hospital, Azienda Socio Sanitaria Territoriale Santi Paolo e Carlo, University of Milan Medical School, Milan, Italy; ^18^Emergency Unit, Azienda Socio Sanitaria Territoriale Santi Paolo e Carlo, Milan, Italy; ^19^Obstetrics & Gynecology, Department of Health Sciences, San Paolo University Hospital, Azienda Socio Sanitaria Territoriale Santi Paolo e Carlo, University of Milan Medical School, Milan, Italy; ^20^Strategic Hospital Management, Azienda Socio Sanitaria Territoriale Santi Paolo e Carlo, Milan, Italy; ^21^Surgery, Department of Health Sciences, San Paolo University Hospital, Azienda Socio Sanitaria Territoriale Santi Paolo e Carlo, University of Milan Medical School, Milan, Italy; ^22^Orthopedy & Traumatology, Department of Health Sciences, San Paolo University Hospital, Azienda Socio Sanitaria Territoriale Santi Paolo e Carlo, University of Milan Medical School, Milan, Italy; ^23^Rehabilitation, Department of Health Sciences, San Paolo University Hospital, Azienda Socio Sanitaria Territoriale Santi Paolo e Carlo, University of Milan Medical School, Milan, Italy; ^24^Surgical Ophthalmology, Department of Health Sciences, San Paolo University Hospital, Azienda Socio Sanitaria Territoriale Santi Paolo e Carlo, University of Milan Medical School, Milan, Italy; ^25^Clinical Psychology, Department of Health Sciences, San Paolo University Hospital, Azienda Socio Sanitaria Territoriale Santi Paolo e Carlo, University of Milan Medical School, Milan, Italy; ^26^Transfusion Unit, Azienda Socio Sanitaria Territoriale Santi Paolo e Carlo, Milan, Italy; ^27^Gastroenterology & Hepatology, Department of Health Sciences, San Paolo University Hospital, Azienda Socio Sanitaria Territoriale Santi Paolo e Carlo, University of Milan Medical School, Milan, Italy; ^28^Respiratory Medicine, Department of Health Sciences, San Paolo University Hospital, Azienda Socio Sanitaria Territoriale Santi Paolo e Carlo, University of Milan Medical School, Milan, Italy

**Keywords:** psychology, gynecology, neurology, pathology, internal medicine, infectious diseases respiratory medicine, COVID-19

## Abstract

In March 2020, northern Italy became the second country worldwide most affected by Covid-19 and the death toll overtook that in China. Hospital staff soon realized that Covid-19 was far more severe than expected from the few data available at that time. The Covid-19 pandemic forced hospitals to adjust to rapidly changing circumstances. We report our experience in a general teaching hospital in Milan, the capital of Lombardy, the most affected area in Italy. First, we briefly describe Lombardy's regional Covid-19-related health organizational changes as well as general hospital reorganization. We also provide a multidisciplinary report of the main clinical, radiological and pathological Covid-19 findings we observed in our patients.

## Introduction

**Figure 1:**
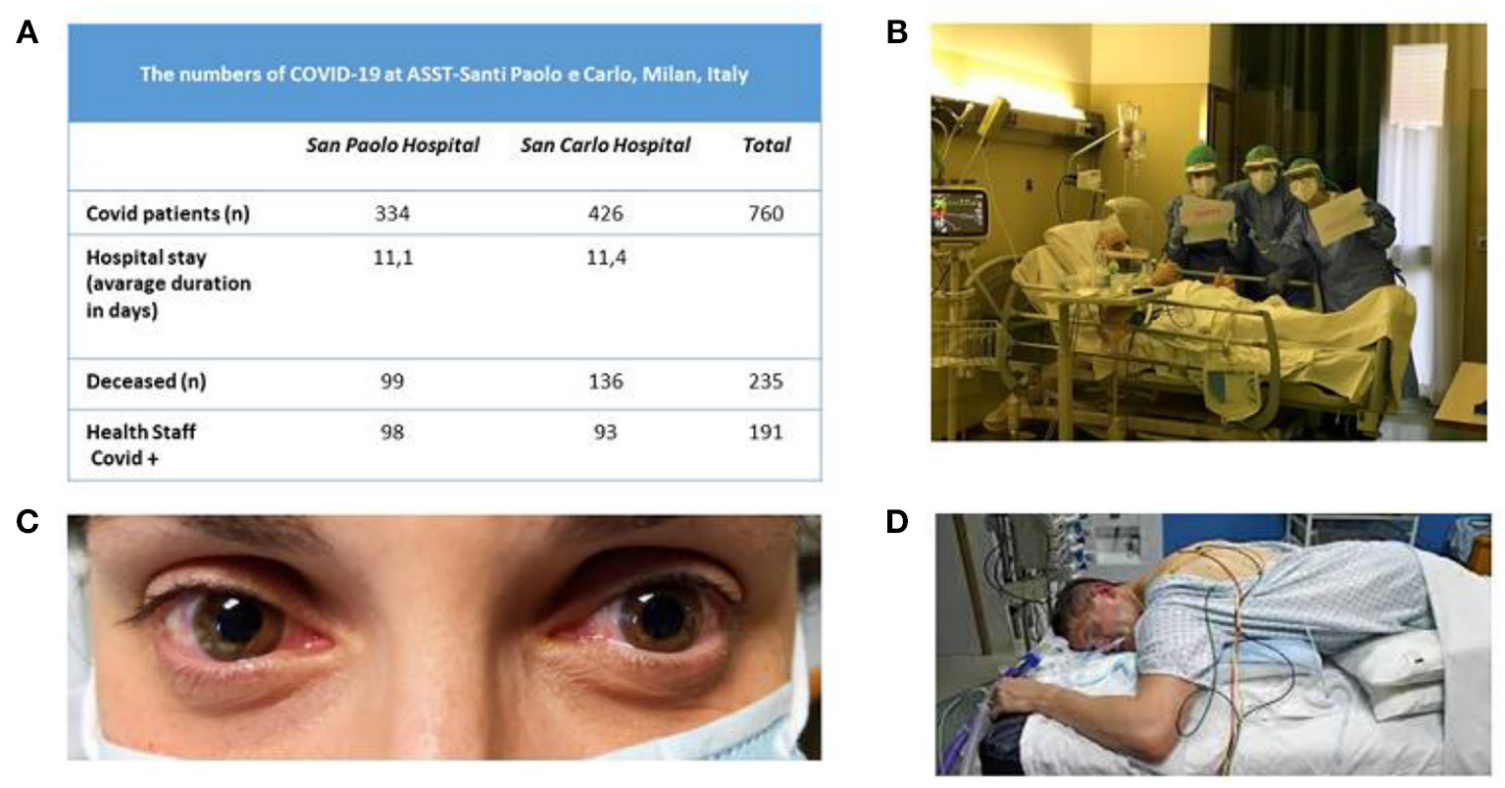
**(A)** Numbers of patients at ASST Santi Paolo e Carlo University Hospital in Milan (from March 1, 2020 to April 16, 2020) during the Covid-19 epidemic. **(B)** Nurses celebrating a patient's birthday at the bedside. **(C)** A representative case of Covid-related conjunctivitis. **(D)** Cycle for positioning a patient in the prone position in the intensive care unit.

In March 2020, northern Italy became the second country worldwide most affected by Covid-19 and the death toll overtook that in China. The epidemic was far more severe than expected from the few data available at that time (Figure 1A). The Covid-19 pandemic forced Italian hospitals to adjust to rapidly changing circumstances.

## Organizational Changes: An Outline

To address the urgent need for physicians, our hospital assigned all doctors to “*Covid teams*.” Each comprised a specialist in infectious diseases (ID), respiratory medicine, internal medicine, and other specialties (including ophthalmology, pathology, maxillofacial surgery, ear-nose-and throat, neurology, general surgery, gynecology, orthopedic surgery, and urology). Covid-related specialists could train others to treat patients alone if the epidemiologic situation worsened or some doctors got sick.

To assist patients with acute illnesses (cardiovascular disease, stroke, oncology, and surgery) Lombardy instituted a medical system with 13 central hospitals (*Hubs*) and 42 peripheral hospitals (*Spokes*). These hospitals created a territorial network and shared protocols that would guarantee appropriate, timely medical environments and ensure patients' safety.

Hospital managements devised specific procedures and made personal protective equipment (PPE) routine in all the wards. Keeping the department running meant implementing preventive measures instructing patients on the importance of self-care (e.g., wearing a mask, hand washing). Hospital directors limited access only to patients, all having their temperature measured and undergoing careful investigation about fever and respiratory symptoms at home. No relatives could enter. Patients had to use gloves and facial masks, a directive that in a delicate setting such as psychiatry, interfered with the therapeutic relationship.

During the Covid-19 outbreak, academic staff made a great effort to maintain teaching by rapidly activating online lessons, seminars, webinars and examinations for medical students and residents. We watched our students through the computer screen, defending the results of their thesis, sitting alone in their bedroom without comfort from cheering relatives and friends, but wearing their best clothes, as if participating in the real ceremony. Residents stopped rotation and flanked the *Covid teams* according to their expertise. Last-year ID, respiratory medicine, internal medicine, and anesthesiology residents were recruited into the medical staff.

Operators had to prolong their working hours and skip their day off. The hospital therefore provided a “decompression room” where staff could go to relieve work pressures and a “muscle reconditioning” space for those who complained of muscle tension.

A powerful tool that is easily accessible to the whole population from children to the elderly, ensures assistance at home for many patients and reduces the danger of infection, is an internet, video or telephone call. Most wards had a call center to answer patients' phone calls and e-mails and reschedule medical appointments. Outpatient activities diminished to urgent patients for all specialties and mainly comprised videotelephony consultations. For instance, remote birth support courses were held through the Zoom platform with interactive lessons involving a midwife and about 20 women at a time. A video was also shot to include a virtual tour of the birth area. The department's Facebook page published information slideshows on preventing coronavirus infection and on how to behave in the later pregnancy stages. Telepsychotherapy also proved useful (1).

Nurses (Figure 1B) have day-by-day been facing difficult situations under psychological pressure. Looking into the eyes of a patient needing oxygen clenching the nurse's hand in the desperate effort to breathe enforces our knowledge that such patients are facing this situation alone. When their shift ends, nurses find it difficult to leave this behind. And equally stressful, upon returning home they have to keep at a distance from others, owing to the risk of transmitting the disease. Hence, they face a high risk of experiencing psychological “scars” that will be hard to heal. In June 2020, voluntary health care professionals (*n* = 264) in our hospital filled in an online questionnaire assessing anxiety, depression and post-traumatic stress disorder to assess their emotional response after the first pandemic wave: 44% had at least one abnormal score in one scale. Among them, most agreed to have a clinical consultation with a psychologist and had an individual or group intervention. The most common complaints were sense of meaningless, issues connected to the moral distress, and fatigue (manuscript in preparation). In a survey conducted in 1,407 hospital health workers in Spain during the Covid-19 pandemic, about a quarter had acute psychological symptoms and about half of the total sample reported physical symptoms (2).

## Clinical Findings: A Glance From the Specialists

**Figure 2:**
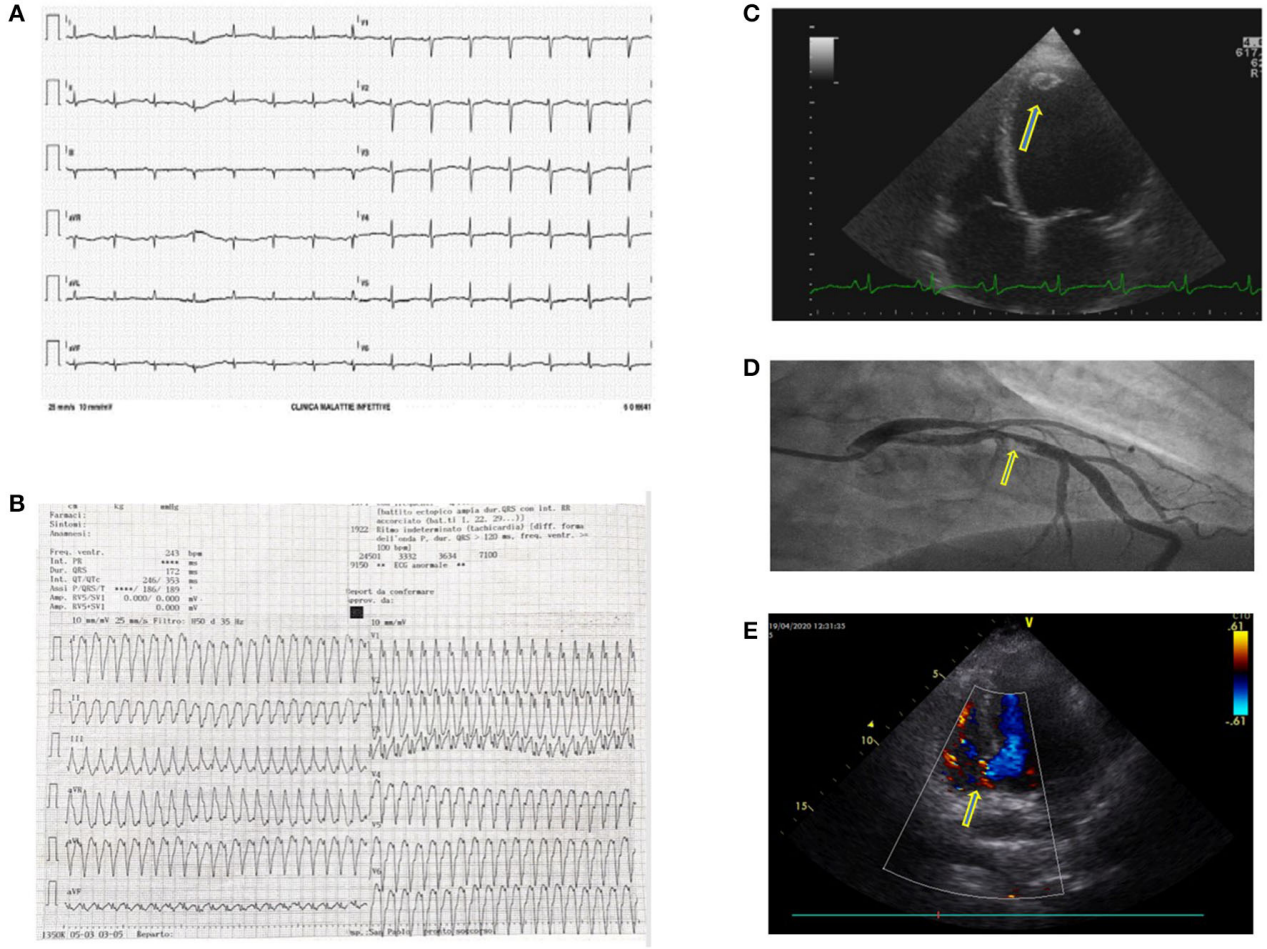
**(A)** Electrocardiogram (ECG) showing a prolonged QTc interval in a Covid-19 patient treated with hydroxychloroquine and azithromycin. These two drugs combined may prolong the QT interval by more than 500 ms and induce arrhythmias. **(B)** Sustained ventricular tachycardia in a Covid-19 patient. The ECG showed frequent malignant ventricular arrhythmias. **(C)** A further Covid-19 related complication was myocarditis probably due to a direct heart muscle infection. This 4D echocardiographic image shows a ventricular dilatation and a concomitant apical thrombus (yellow arrow). **(D)** Coronary thrombosis in a Covid-19 patient with an acute coronary syndrome. In Covid-19 patients, we frequently found coronary vessel thrombosis obstructing the coronary artery. In this patient, the ECG clearly shows a blood clot obstructing the interventricular coronary artery (yellow arrow). **(E)** Interventricular septum rupture (yellow arrow, basal septum rupture with shunt) in a Covid-19 patient who arrived late in the coronary unit during S-T elevation myocardial infarction.

Our hospital was one of the three cardiological Hubs in Milan. During the first month, *cardiologists* treated 32 patients presenting with ST-segment elevation myocardial infarction, nine were Covid-19+. During the first week only 3 were dispatched to our center, whereas thereafter numbers progressively increased. Though we were aware of cardiological complications (3–6), we frequently observed acute coronary syndromes, coronary thrombosis in young patients, syncope, atrioventricular block, and myocarditis. We had one case of heart rupture. Overall, patients arrived in the emergency room late (Figure 2). Recent data from a systematic review published after the first epidemic wave report a prevalence of cardiologic complications ranging from <1 to 44% (7). This wide variability arises from the features of the population examined (age, prevalence of comorbidity).

*Psychologists* also helped patients suffering from anxiety and depression, and hospitalized patients' relatives. Patients who recover and go home may experience survival guilt. Because a phone call helps grief and mourning, psychologists have been phoning the relatives' next of kin after a patient dies. The long-term consequences of such a dramatic event need to be carefully assessed: Post-traumatic stress disorder (PTSD) may persist for years (8–10).

Though Covid-related skin abnormalities are now increasingly emerging (11), our *dermatologists* at the outbreak observed no Covid-related complications. Some healthcare workers had hand eczema possibly related to frequent hand washing and to gloves. Later publications reported skin findings including chilblain-like lesions in 40.2%, maculopapular lesions (22.7%), urticarial lesions (8.9%), vesicular lesions (6.4%), necrotic lesions (28, 2.8%), and other rashes and skin lesions (19.8%). Other skin findings described included pain, burning and itch. The reported prevalence of Covid-related skin manifestations varied from <1% to 20.45% (12–14).

Considering that *dialysis* (15) cannot wait for virology, Covid-19 patients must be hospitalized and undergo dialysis in the ID ward or ICU with mobile dialytic devices. Some patients enter the ICU with acute kidney injury. About 15% of ICU patients develop acute kidney injury whereas 5% of all patients require renal replacement therapy. Renal failure is acute, owing to tubulo-interstitial damage, and often reversible. In patients with chronic kidney disease renal function might worsen. In their study published this year, Kellum et al. (16) report that, like other pneumonias requiring hospital admission, acute kidney injury in patients hospitalized with Covid-19 is as high as 43%, and is strongly associated with mortality. In contrast, mortality is lower in Covid-19 patients without acute kidney injury. Similarly, Jager et al. (17) assessed 28-day mortality after Covid-19 diagnosis in European kidney replacement therapy patients between February 1, 2020 and April 30, 2020 encompassing 4,298 cases. After a 28-day follow-up, they report a 20.0% Covid-related mortality of 20.0% in 3,285 patients undergoing dialysis, and 19.9% in 1,013 transplant recipients. They identified differences in Covid-19 mortality across countries, and an increased mortality risk in older patients receiving kidney replacement therapy and men receiving dialysis. In kidney transplant recipients older than 75 years, 44.3% did not survive Covid-19. The mortality risk higher in transplant recipients than in dialysis patients. Hence, Covid-19 remarkably increases mortality in patients receiving kidney replacement therapy. This population therefore needs a high protection level from the disease.

In the *emergency department (ED)* the major need was to isolate Covid-19 “suspected” or “positive” patients from others. We isolated them in the negative-pressure rooms and in another unused ED area until the Covid-19 swab results were available. Patients with low blood-oxygen levels received continuous positive airway pressure (CPAP) and bilevel (BIPAP). The ED had to provide non-invasive ventilation for up to 15 patients at the same time. Typical symptoms were malaise, mild fever (usually up to 38°C), dry cough, nasal and conjunctival congestion, dysgeusia, and dysosmia; few patients had diarrhea (18, 19). Patients with pneumonia typically had respiratory symptoms (from shortness of breath to true respiratory distress) tachypnea and rales. Typical laboratory findings were lymphopenia, high C-reactive protein, and lactate dehydrogenase plasma levels. Many patients also had increased plasma D-Dimer. Other emergency admissions dropped dramatically. The study by Ojetti et al. reported a 37.6% overall reduction in ED admissions in 2020 compared with the same period in 2019 (20). Admissions for cardio-thoracic, gastroenterological, urological, otolaryngologic or ophthalmologic, and traumatological diseases decreased. The study conducted in Rome, Italy, reports that acute neurological conditions slightly decreased whereas oncology admissions remained stable. Presentations for infectious diseases were 30% in 2020, compared with 5% in 2018 and 6% in 2019. In 2020, the admission rate was 42.9% compared with 27.7%, and 26.4% in previous years. Decreased ED admissions during the Covid-19 epidemic could reflect a fear of infection. Covid-19 possibly also taught us to use the ED better. But worrying numbers concerned the decreased cardiology and neurology admissions: patients delayed medical consultations, sometimes incurring lethal consequences, just because they feared Covid-19, ultimately leading to avoidable morbidity and mortality. Admissions for stroke also dramatically decreased compared with the same period in the previous year. In their study published this year, Sharma et al. (21) report a decrease up to 46% in ED stroke alerts during the pandemic.

The *gastroenterology and hepatology* unit changed into a Covid ward. A small “clean” area remained for patients with cirrhosis, hepatocellular carcinoma, and exacerbated inflammatory bowel disease. Covid-19 frequently involves the liver causing increased blood alanine aminotransferase (22). Concomitant with respiratory symptoms or appearing alone, gastrointestinal manifestations primarily involve diarrhea (23), nausea and vomiting. In their study, Sulaiman et al. (24), describe isolated gastrointestinal symptoms in 23.6% of the patients; 44.3% of cases had respiratory symptoms alone, whereas 32.1% had respiratory and gastroenterological symptoms combined. Patients with gastroenterological disturbances alone have less severe disease. Patients with isolated gastroenterological symptoms have no mortality though why this is so remains unclear.

*Gynecologic* services limited to oncology and emergency. Pregnant women entering the clinics were screened with a 13-item questionnaire translated into 12 languages. We converted the day surgery obstetrics and gynecology ward into a Covid-19 ward. The day hospital for legal pregnancy terminations maintained its activity. No obstetrics or gynecologist specialist observed specific Covid-19-correlated complications. In their study earlier this year, Afsahr et al. assessed the clinical presentation of Covid-19 in pregnant or recently pregnant woman (25).

The most frequent symptoms in patients positive for SARS-CoV-2 were cough (20%), sore throat (16%), body aches (12%), and fever (12%). Though symptoms disappeared on average in about 5 weeks, in 25% of participants symptoms persisted for 2 months or more.

The ICU in our hospital almost exclusively admitted Covid-19 patients. Operating rooms accommodated additional ICU beds. ICU patients needed remarkably high care levels including repeated pronation cycles (Figure 1D) and in one case extracorporeal circulation. The ICU also reserved few beds for emergency and oncologic surgery in non-Covid-19 patients. Many patients admitted to our ICU suffered from acute respiratory distress syndrome (ARDS). Soon after admittance, they received moderate positive end expiratory pressure (PEEP) levels with sedation. Patients who did not recover after 5–7 days were tracheostomized and began weaning. When intubation ended, several patients had hiccups and respiratory dyssynergia probably due to the bulbar damage (26). According to an Italian multicenter study (27) including more than 1,400 charts for patients with Covid-19, 16.7% required ICU admission, more frequently man and with comorbidities (74%). The most relevant risk factors for ICU admission were obesity, kidney failure and arterial hypertension.

Our *ID* activity took place partly directly in the ED to plan initial medical care (28). The ID negative-pressure rooms changed into subacute ICUs to provide non-invasive ventilation. An extremely important task is to provide oxygen supplementation and to ask the ICU specialists as early as possible to supply invasive ventilation if needed. Those at highest risk of poor outcome are the elderly but the young also need careful attention because they occasionally undergo a turbulent course. Patients may take months to return negative. A critical issue concerned the 5–10% patients with pneumonia requiring O_2_ therapy with a chest CT scan typical for Covid-19 but no reactivity to SARS CoV2 polymerase chain reaction (PCR) tested by nasopharyngeal swab (NPS). As we explain in the foregoing text for some pediatric patients we have seen, also in patients whose clinical presentation resembles Covid-19 manifestations, physicians should always rule out other treatable infections (such as infective endocarditis) even in patients with a positive nasopharyngeal swab (29).

**Figure 3:**
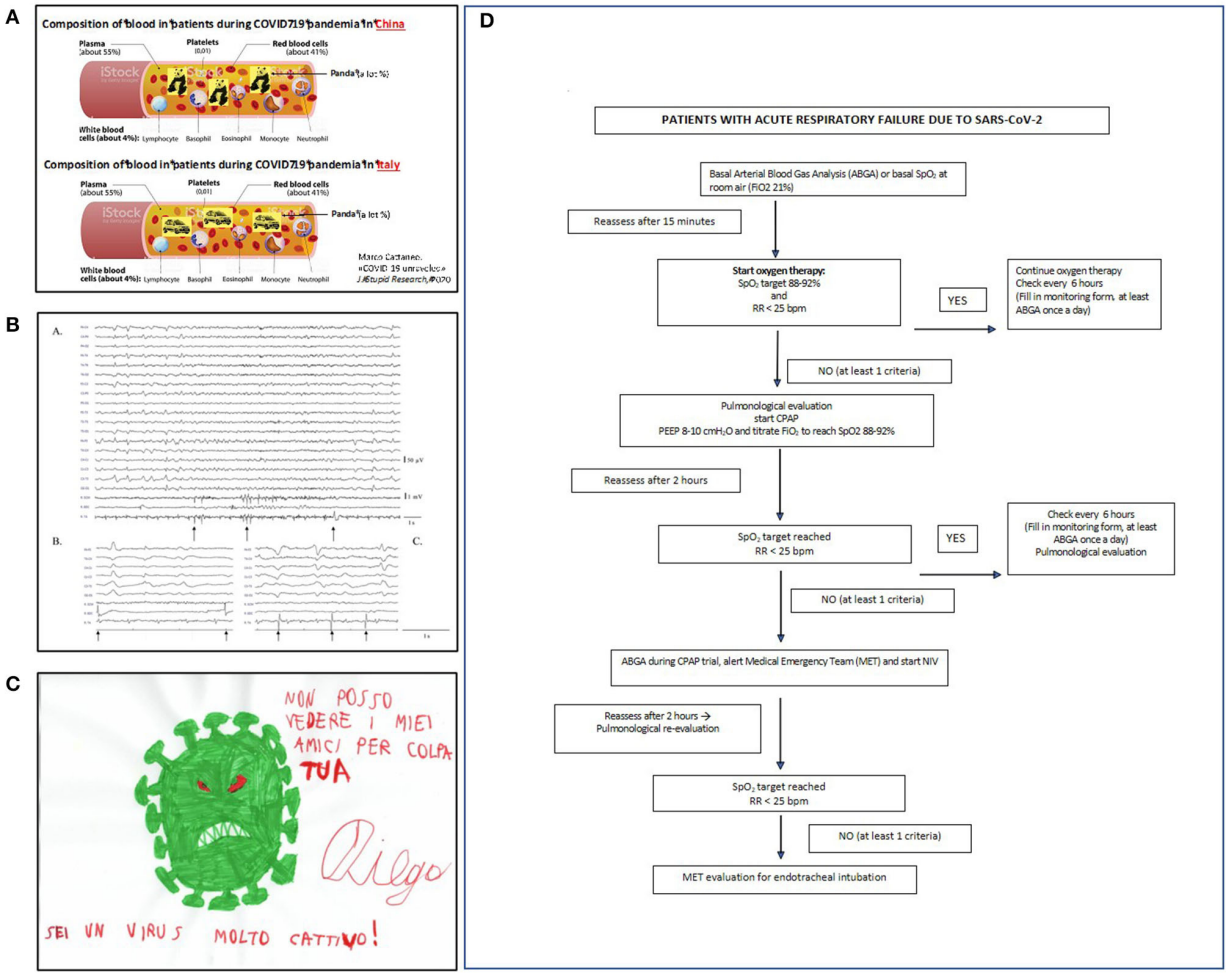
**(A)** An “alternative” pathophysiological hypothesis for Covid-19 pandemia developed at the department of internal medicine. Amusing moments helped staff to face the disaster (modified from https://www.news-medical.net/health/Blood-Plasma-Components-and-Function-(Italian).aspx). **(B)** (a) Some Covid-19 patients had myoclonus with a typical electroencephalographic (EEG) pattern showing both periodic lateralized epileptiform discharges (PLEDs) and bilateral PLEDs (bi-PLEDs), predominantly involving the left hemisphere, mainly recorded in the parasagittal and midline regions. (b,c) Black arrows indicate myoclonic jerks, without any correlation with periodic lateralized discharges, nor with a prominent proximal-to-distal gradient of appearance (surface electromyography recorded from the right *sternocleidomastoid, extensor carpi radialis longus*, and *tibialis anterior* muscles). **(C)** The virus as seen by an autistic child aged 8. In children with psychiatric disorders, Covid-19 remarkably influenced their thought and mood. **(D)** The algorithm for ventilation used in our patients (PEEP, positive end-expiratory pressure; SpO_2_, pulse oximetry; FiO_2_, fractional inspiration of oxygen; NiV, non-invasive ventilation; CPAP, continuous positive airway pressure).

Many attribute the high incidence of acute respiratory worsening to pulmonary embolism (30) and suggest that high-dose heparin should administer for thromboprohylaxis. However, our *internal medicine* unit (Figure 3A) used standard low-dose low-molecular weight heparin: none of the more than 300 patients hospitalized in our wards had symptomatic deep vein thrombosis (DVT), while systematic ultrasonographic testing failed to detect asymptomatic proximal DVT in 64 patients. We hypothesized that the described pulmonary vascular occlusions are accounted for by pulmonary artery thrombosis, rather than pulmonary embolism (31), considering the low reported incidence of DVT, which represent the origin of pulmonary emboli (30). A recent meta-analysis by our internal medicine group compared the frequencies of DVT and “pulmonary artery occlusion” in patients with Covid-19 with those observed in previously reported patients without Covid-19: all had been under thromboprophylaxis with low-dose heparin (32). In non-ICU wards, the frequency of DVT was low and similar in Covid-19 (4.57%) and non-Covid-19 patients (3.64%), suggesting that the risk of DVT is not higher in Covid-19 than in non-Covid-19 patients. In contrast the frequency of “pulmonary artery occlusions” was much higher in Covid-19 patients (2.55%) than in non-Covid-19 patients (0.11%), thus supporting our hypothesis that most pulmonary artery occlusions in Covid-19 are attributable to pulmonary artery thrombosis, rather than pulmonary emboli (32).

The *neurology* unit became a Covid ward. For the first 2 weeks no patients with acute neurological problems presented to the ED. Neurological signs or symptoms related to central nervous system invasion (26) were confusion, delirium, seizures, impaired smell and taste, muscle pain, and headache (Figure 3B). Covid-19 survivors often manifest polyneuropathy, severe generalized muscle atrophy, and attention and memory deficits. Their long-term outcome is unknown. In their study assessing the occurrence of neurological symptoms in Covid-19 patients, by distributing a survey to physicians involved in their care, Campiglio and Priori found that 87.3% of practitioners observed neurological symptoms. In most cases these were mild and non-specific, but in few patients, they were severe (33). According to Lechien et al., 85.6% of Covid-19 patients report olfactory symptoms and 88% gustatory dysfunctions. In 11.8% of the cases, olfactory dysfunction (OD) appeared before the other symptoms. Women are significantly more affected by olfactory and gustatory dysfunctions than men (*p* = 0.001) (34).

To prevent hospital-acquired Covid-19 infections, the *Occupational Health* unit activated specific surveillance of our healthcare workers (HCWs). “Close contacts” of Covid-19 infected persons were identified and authorized to work until molecular test results wearing PPE (surgical mask) and maintaining social distancing even at home. In case of positive results, they entered 14-days quarantine if asymptomatic, whilst symptomatic prolonged quarantine until 10 days after the end of symptoms. Thereafter, only workers in whom two consecutive NPS gave negative results were readmitted. This approach (35) brought to the result that from March to June 2020, the epidemics involved only about the 4% of the hospital healthcare staff, whilst in other structures of the same Region, the incidence reached levels of 6–7%, with peaks exceeding 15%. A second interesting finding of our approach was that only 10% of our hospital workers who had positive NPS were symptomatic: most complained only of abnormal taste and smell (36). In line with our infection rate, a later large Italian study by Porru et al. (37) identified positive nasopharyngeal swabs in 4% of Italian health workers. Another study on healthcare workers found in 17,971 samples for SARS-CoV-2 antibody testing an overall seroprevalence of 3.4% (confidence interval: 2.5–3.8%) (38). Emergency departments had the highest seroprevalence (29.7%), whereas departments without patients or with limited patient contact had the lowest (2.2%). Hence healthcare workers should probably undergo regular testing for SARS-CoV-2. This the approach adopted by our Occupational Health Unit during the so called “second wave” of the epidemics. A serologic retrospective study in in healthcare workers in our hospital revealed that individuals with IgM and IgG were 14.4 and 7.4%, respectively with no relationship was between exposure to Covid-19 patients and serological status (39).

*Oncologists* maintained ward and outpatient activity. The incidence and severity of Covid-19 infection appeared initially higher in cancer patients (40). In symptomatic patients with positive NPS, we suspended chemotherapy for at least 2 weeks, and in those with symptomatic infections until complete resolution. Caution is essential in giving drugs highly toxic to lungs, such as bleomycin, cyclophosphamide, methotrexate, and immunotherapy. Covid-19-free patients can continue their chemotherapy. Overall, a French study by Helissey et al. suggested that Covid-19 negatively influenced oncological patients: 47.6% of the outpatients received modified patient care, 24% of scheduled surgeries were postponed, or took place without perioperative chemotherapy, 18.4% followed a hypo-fractioned schedule, and 57% had an adaptive systemic protocol (stopped,0 oral protocol, and spacing between treatments) (41). About 70% of physicians used telemedicine. During the study, 67% of the physicians reported that taking care of their patients caused no distress. About 2/3 of physicians worried about how lockdown would affect future patient care. Oncologic patients are considered at risk for severe Covid-19 given that malignant diseases and chemotherapy weaken the immune response. But, in contrast with earlier studies, Hempel et al. who tested 1,227 oncologic patients for Covid-19 found 6.4% of them positive (42). Only one positive patient experienced severe Covid-19 with pneumonia. None of the asymptomatic patients had Covid-19 complications during the oncologic treatment. These findings argue against the hypothesis that oncologic patients are vulnerable to Covid-19 and points out that they can safely receive cancer therapies. Last, the relatively low incidence of Covid-19 symptoms in patients receiving chemotherapy and other immunosuppressive drugs such as glucocorticoids suggests that immunosuppression might reduce Covid-19 severity.

**Figure 4:**
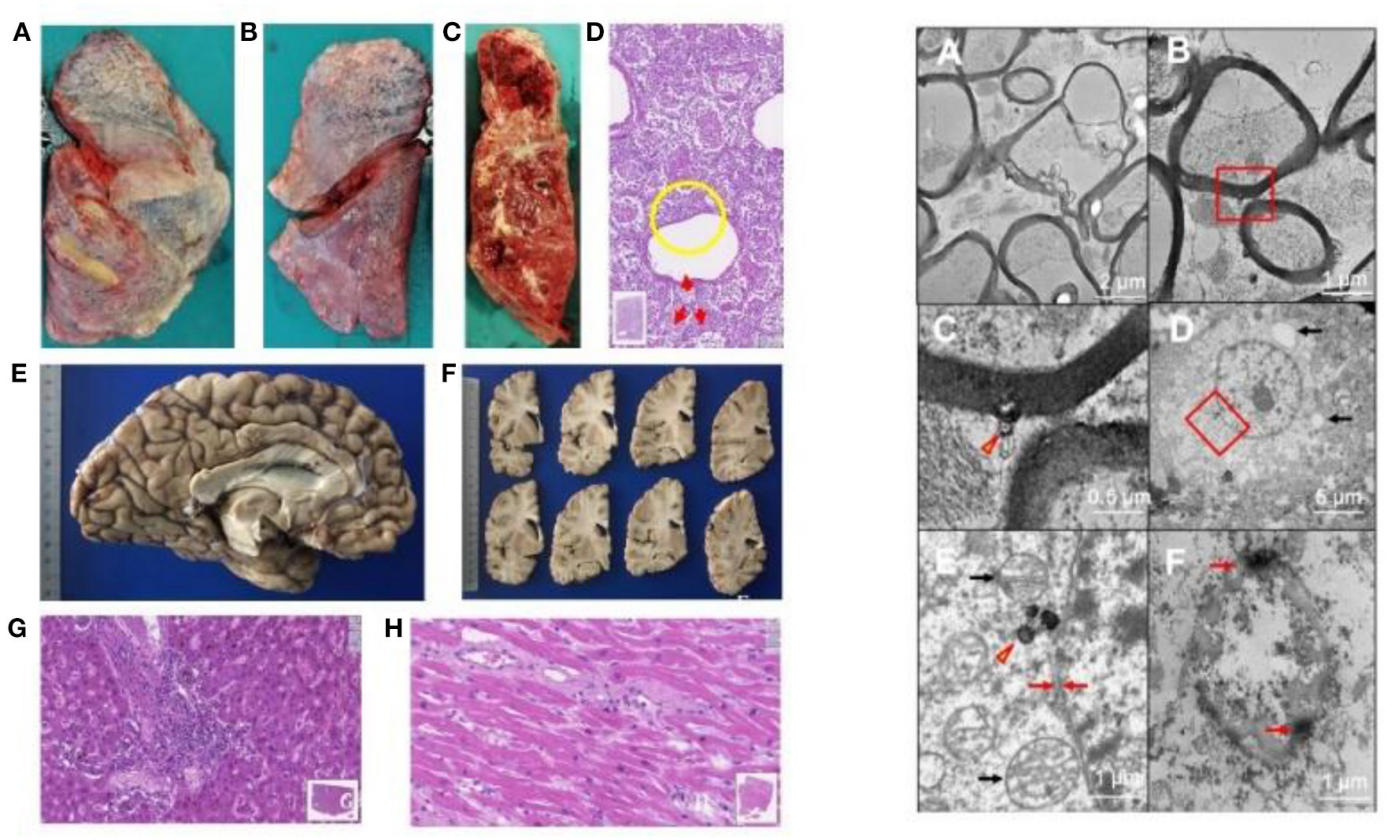
LEFT. Autopsy findings in Covid-19 positive patients. Patients who died underwent autopsy 2–3 h after death, ascertained by a continuous 20-min electrocardiographic recording. **(A–C)** Lung findings at autopsy. Gross autopsy examination shows that the lungs have increased consistency but reduced volume. The lung parenchyma on the cut surface appears dry: squeezing produces scarce aerated blood. The lung parenchyma varies in color from bright red to grayish, with hemorrhagic areas of various sizes [**(A)** right lung, outer surface. **(B)** Left lung: external surface. **(C)** Left lung: cut surface of the parenchyma]. **(D)** Histologically, the lung alveolar cavities display abundant cellularity and fibrin aggregates (one in the yellow circle). The red arrows indicate in areas surrounding the bronchiolar walls amorphous material suggesting hyaline pulmonary membranes (E-E; 11.5x magnification; Hamamatsu NanoZoomer digital slide). **(E,F)** Left cerebral hemisphere without macroscopically evident pathological alterations. **(G)** Liver. The image highlights a portal septum with marked inflammatory infiltration. Inflammatory cells attack the adjacent hepatocyte plate and can also be seen within the sinusoidal spaces. The liver image also shows changes indicating biliary stasis (E-E; 19.3x magnification; Hamamatsu NanoZoomer digital slide). **(H)** Left ventricular myocardium. The gap between the myocytes contains mild but widespread inflammatory infiltration (E-E; 33.5x; Hamamatsu NanoZoomer digital slide). Right: Involvement of the nervous system in SARS-CoV-2 infection on transmission electronic microscopy. Ultrastructure in the medulla oblongata **(A–C)**; gyrus rectus **(D,E)**; and olfactory nerve **(F)**. **(A)** Marked axonal damage involving the medulla oblongata, with irregular axonal swelling and disordered myelin sheath. The damage appears widespread. **(B)** A viral particle (red box) is observed in the periaxonal matrix near the outer surface of a myelin sheath. **(C)** Magnification of the red box area in **(B)** the spherical particle (~98 nm) has a crown shape with a dense inner core and electron-dense periphery with small external projections. The center of the particle contains a small roundish electron-dense structure compatible with that of SARS-CoV-2. **(D)** The image shows a neuron in the gyrus rectus, as demonstrated also by a nucleolus in the center of the euchromatic nucleus; autophagy phenomena in the cytoplasm (arrows) suggest cell damage. **(E)** Magnification of the red box area in **(D)**, showing a viral-like particle measuring 160 nm (arrowhead). Black arrows indicate two well preserved mitochondria; the red arrows show the neuron's typical double nuclear envelope. The well-preserved ultrastructural features of these organelles demonstrate adequate collection and fixation methods and suggest that the tissue damage is related to the viral infection. **(F)** Severe tissue damage in the olfactory nerve: the oval structure is difficult to identify and is characterized by extensive autophagy phenomena with markedly electron-dense peripheral aggregates (arrows) (Images by Unitech NO LIMITS).

**Figure 5:**
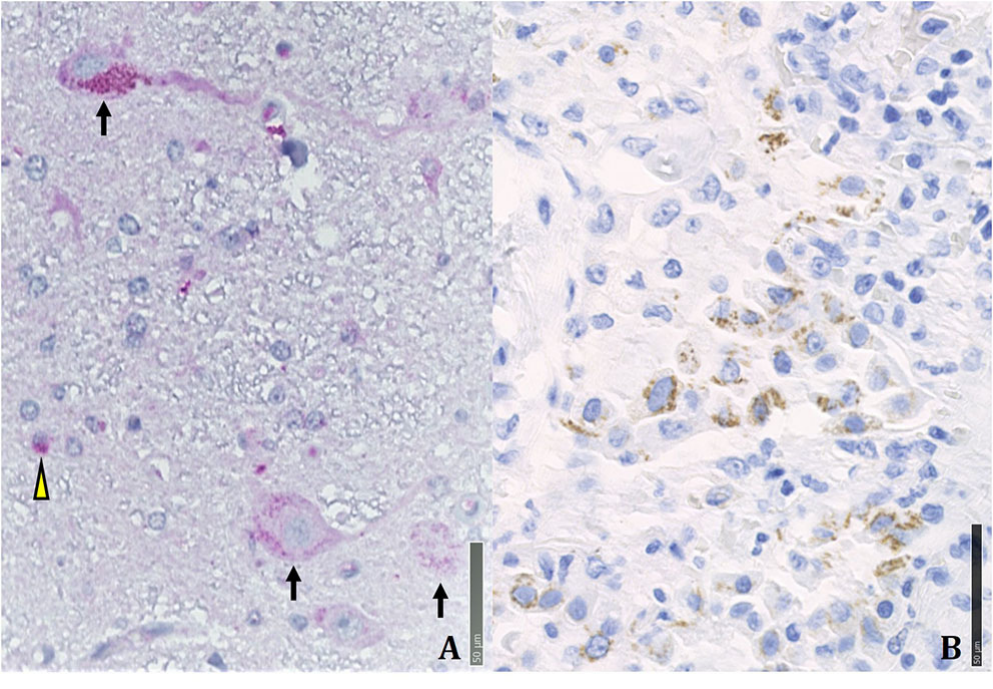
Immunohistochemical positivity for SARS-CoV-2 in autoptical tissues. Immunohistochemistry examination was performed using an automated immunostainer (DAKO OMNIS). **(A)** Brain. The red granule in the cytoplasm of neurons (arrows) and glial cells (arrowheads) indicates the positivity for nuclear protein (NP) of the virus. **(B)** Lung. Widespread cellular positivity (brown granulia) for viral spike protein. Positive cells are largely macrophages and pneumocytes. [**A**: SARS-CoV-2 (2019-nCoV) Nucleoprotein/NP Antibody, Rabbit MAb; Sino Biological; 1:800, antigen retrieval low; detection with DAB. **B**: SARS-CoV / SARS-CoV-2 (COVID-19) spike antibody [1A9] Cat No. GTX632604; GeneTex; 1:100, antigen retrieval high; detection with Magenta].

In the *pathology unit*, overall, diagnostic procedures decreased by some 50%. The most frequent autopsy finding (Figures 4, 5) was septal lung damage involving all the lobes (fibrosis) and pulmonary alveoli (engulfed by inflammatory cells and fibrin).

Many patients had multiple vascular thrombosis. The SARS-CoV-2 infection also produces multiple organ endothelial damage and frequently myocarditis, liver inflammation and nervous system damage. We found the virus in the olfactory system and in the medulla oblongata (26). A review of 28 papers by Maiese et al. on a total of 341 cases showed alveolar damage with hyaline membrane formation and microthrombi in pulmonary vessels (43). In a recent autopsy study by Schurink et al. in SARS-CoV-2, multiple organs had infected cells, especially in the lungs, but during the disease course these decreased (44). SARS-CoV-2-positive tissues also included heart, kidneys, and gastrointestinal tract. A strong inflammatory response involved the lungs, heart, liver, kidneys, and brain. In the brain, extensive inflammation was seen in the olfactory bulbs and medulla oblongata. Many tissues contained thrombi and neutrophilic plugs especially in the late disease stage course after 3 weeks.

The *pediatric* unit was kept open. Children have milder clinical features than adults (45, 46). Those with comorbidities may need intensive care (47). Half of younger patients come with respiratory symptoms. Despite suggestive clinical features and a positive NPS, the remaining Covid-19 positive children had alternative diagnoses, events that pediatricians should always exclude. We observed no case of Kawasaki syndrome. In their study involving 203 SARS-CoV-2-infected children Maltezou et al. observed that 54.7% were asymptomatic (48). Although 45.3% had Covid-19 only 26.1% were hospitalized. Infants <1 year had the disease more frequently than older children. In 74.2% of children the infection originated from a household member. SARS-CoV-2 infection during childhood seems mainly asymptomatic or mild.

Our *pediatric neuropsychiatry unit and epilepsy center* maintained only urgent visits. The remaining outpatient clinics shifted to telehealth [(49, 50); Figure 3C]. Probably because quarantine reduced external stressors with decreased accessibility to recreational drugs and forced family living, decompensation episodes in adolescents with psychiatric diagnoses decreased. The lockdown in some patients increased addictive behaviors for videogames. The social isolation changed daily routines, especially in children. Among the measures intended to reduce viral spread, most schools closed, canceled classes, and moved to home-based or online learning to encourage and adhere to social distancing guidelines. Moving away from physical classes completely distorted the lives of students and their families with implications for children's mental health. Whether these changes will affect adolescent developing brains remains unclear (51). The psychological stress in general population caused by the lockdown might also have influenced the more fragile adolescent (52).

General *psychiatric* outpatient services were dedicated to urgent visits and to patients needing periodic drug administration (53, 54). Most patients had regular telephone updates with staff. During the first 3 weeks, the emergency room and the wards admitted fewer acute psychiatric patients than usual. Acute severe psychiatric patients positive and asymptomatic for Covid-19 entered a dedicated ward. But respecting the required hygiene norms proved difficult. In future months, psychiatrists foresee increased rates for depression and anxiety syndromes. About 11% of Covid-19 cases have delirium (55). These are older patients with neuropsychiatric comorbidities and worse respiratory conditions.

**Figure 6:**
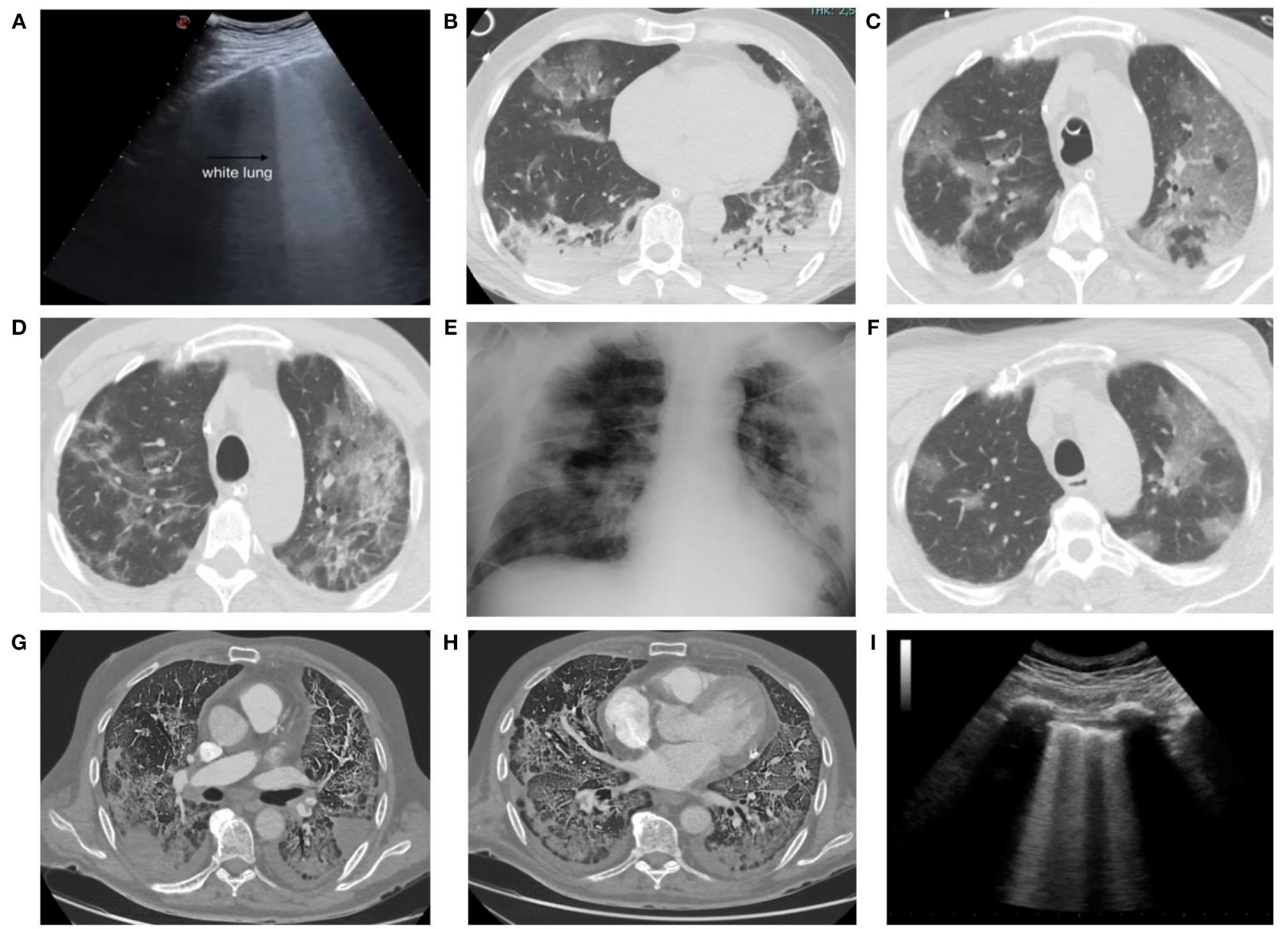
A 55-year-old man admitted to our emergency department with signs and symptoms of respiratory infection underwent chest ultrasound (US) **(A)**, showing white lung areas, then confirmed at computed tomography (CT) examination **(B)** which showed multiple bilateral subpleural areas of ground glass opacity (GGO) in the upper lobes. CT scans obtained 48 h after admission showed the multiple GGO areas increased in number and extension **(C)** and the development of pulmonary consolidations in the basal posterior and lateral segments of both lower lobes **(D)**. Chest x-ray obtained 9 days after admission **(E)** showed multiple coalescent pulmonary consolidations involving both lungs, mainly in the periphery. CT scans obtained 13 days after admission showed the GGOs in the upper lobes now partly regressed **(F)**. CT scan in a 75-year-old man, with severe lung involvement, secondary to SARS-CoV-2 infection, showing pulmonary thromboembolic disease involving segmental arteries in the left **(G)** and right **(H)** lower lobes with triangular shaped subpleural consolidations (pulmonary infarcts). A 68-year-old man admitted to our emergency department with signs and symptoms of respiratory infection then resulted Covid+. Chest US showed an interstitial syndrome, characterized by more than three B lines per longitudinal field **(I)**.

Key points for *radiological* CT diagnosis (Figure 6) are multiple ground-glass opacities (GGO), crazy-paving pattern, consolidation shadows, mainly distributed in the bilateral peripheral and subpleural lung areas. The CT decontamination required after scanning patients interrupts radiological service availability and suggests minimizing the risk of cross infection with a bedside chest X-ray. Portable chest radiography is therefore preferred for follow-up. Ultrasounds are quick, can be done in the emergency room, and accurately detect subpleural thickenings. In anosmic patients, magnetic resonance imaging (MRI) disclosed reduced olfactory bulb volumes. Cardiac MRI detected acute myopericarditis and systolic dysfunction.

Angio-CT scan detects pulmonary thromboembolism. In their radiological study, Zhao et al. report as the most frequent findings, GGO in 85.7% and lung vascular enlargement in 78.6% of the cases. In these lesions, 64.3% of the margins were uneasily differentiated. The authors conclude that the viral load inversely correlates with an uneasily differentiated lesion margin on the first CT scan images (56). Despite this conclusion, Salameh et al. conclude that chest CT is sensitive but not specific for Covid-19 in suspected Covid-19 patients (57). Hence, CT cannot distinguish SARS-CoV-2 infection from other respiratory diseases. This low specificity could also reflect the poor sensitivity of the reference standard (RT-PCR), because CT could potentially be more sensitive than RT-PCR in some cases. Chest X-ray and chest ultrasound findings need to be cautiously interpreted.

The *rehabilitation* unit reduced the number of beds but opened a new ward for monitoring Covid-19 patients. Physiotherapy activities remained available only for urgent care and for traumatized patients. ICU patients had a hypomobility syndrome and underwent early passive mobilization. In the post-acute stage, some patients had critical illness polyneuromyopathy and entrapment neuropathies. When their respiratory function improves, patients complain of fatigue (58). Severely ill patients had rather long recovery times (often more than 12 weeks). Given the numerous patients recovering from Covid-19, much help can come from delivering tele-rehabilitation.

The *respiratory medicine* unit was entirely dedicated to Covid-19 and 60 new beds were created for sub-intensive care. Pulmonary function tests and outpatient visits were limited to urgent requests. Patients with Covid-19 pneumonia were mostly men and >40 years old. Frequent symptoms were fever, dry cough, and dyspnea. When the respiratory rate is >25 breaths/minute or pO2 is <70 mmHg or oxygen saturation is <92%, or all three are combined, positive-pressure ventilation (with helmets or masks) is obligatory before intubation (Figure 3D). High BMI, hypertension, diabetes, and symptom duration predict a poor prognosis.

*Surgical specialties* were unified into a single “Covid free” Unit. The only services maintained were emergency, and oncological surgery. Patients who required an emergency surgical intervention but had positive Covid-19 NPS, went to the Covid units. Surgeons redefined surgical guidelines (59). In the operating theater, standard protocols were defined for PPE and gas aspiration mainly in laparoscopy. During the initial emergency, some patients who underwent elective surgery developed Covid-19. Though basic *maxillofacial* surgical activity was maintained (60), no patients had specific Covid-related maxillofacial pathologies. In contrast, the incidence of facial fractures and cervico-facial cellulitis of odontogenic origin underwent a major decrease. Whereas, in March and April 2019 and 2018 one patient per week needed therapy for cellulitis, between March and April 2020s no patients had cellulitis (61).

*Ophthalmology* clinics were limited to emergencies, surgical patients' follow-up, and intravitreal injections for maculopathies. The only surgical procedures permitted were those for glaucoma and retinal detachments. We saw few cases of conjunctivitis (Figure 1C), a condition associated with Covid-19 (62). A recent Italian study reported ocular disorders in 26.2% patients. Covid-19 patients receiving CPAP have ocular abnormalities more frequently than those without CPAP (63).

*Orthopedic* activity concentrated on urgent fractures. Traumatology care dropped but 3 weeks after the lockdown rose again. If negative, patients were hospitalized in the Covid-free surgical ward and followed the standard treatment protocol (64). Of major concern 30-day mortality among hip fracture patients during the first 30 days of the pandemic increased. A positive Covid-19 test in patients with hip fractures is associated with a 2.4-fold increase in the risk of 30-day mortality (65).

*Otorhinolaryngology (ENT)* specialists reorganized care (66) yet kept the service running, progressively limiting outpatient procedures to emergencies. The ENT ward was closed. Basic surgical activity was maintained. In Covid-19 patients, ENT specialists treated nose bleeds and did tracheostomies in ICU patients who had a complex neck anatomy. Patients with lung cancer underwent *thoracic surgery* within 30 days after the diagnosis (41). Lung cancer referrals will presumably increase when the Covid-19 emergency finishes. Whether the delay, and indirectly the Covid-19 pandemic, will influence these cancer patients' outcome remains unclear. A recent collaborative study on 115 patients with lung cancer (adenocarcinoma 66%, cT1 62%, cN0 82%) reports that in the first month after surgery 5% of patients were diagnosed with Covid-19 (67). Positive patients underwent surgery during the first month of the pandemic and were more frequently receiving corticosteroids preoperatively. Post-operative Covid-19 implied a higher readmission rate but no change in morbidity or mortality. At the *transfusion unit* the need for blood decreased by some 30%. Patients in oral anticoagulation and those on vitamin K antagonists underwent testing in external laboratories and phone or email counseling (68). When planning future services, we need to remember that despite having significant systemic disease, Covid-19 patients rarely require transfusion (69). A possible therapeutic approach is convalescent plasma collected through plasmapheresis from donors who have recovered from Covid-19 (70, 71). In their study on patients treated with convalescent plasma, Ibrahim et al. reported that 63% recovered and were discharged, and 37% died. Patients treated early had a lower mortality rate and shorter hospital admission. Convalescent plasma infusion caused no adverse effects (72).

## Shadows, Lights, and Lessons From COVID

A key point is that, because no data are available from China, and given that Italy was the first European country involved by the pandemic, the organizational changes we report lacked an organizational analysis and their effectiveness remained unevaluated, compared with other possible interventions. When the present article was written (April–May 2020) at the time of the facts reported that so rapidly invested the northern Italy, no model was available and the decisions depended on the following factors: the hospital's specific competences, the technical ward supplies, the need within days to divide the whole hospital into “clean” and “dirty” areas, the stormy epidemics, the need to support other hospitals in the neighboring red areas, the need to guarantee emergency management also for non-Covid-19 patients and last but most important, the market shortage in the first 3–4 weeks of devices for the medical staff's personal protection.

In judging our model's effectiveness we underline that our organization never implied that doctors in the ICUs had to decide which patients to save, mortality 29.6% (Figure 1) corresponded to the average in the Lombardy area (and few staff members became infected (4%) again in line with values reported in Italy. A Spanish study reported 11% of infected staff (73). Mortality in other Italian hospitals in northern Italy varied between 43.6% (74) and 23.2% (75). In other hospitals in the Lombardy area, mortality ranged from 14.4 to 36.7% (75–80). Including our data, during the first epidemic wave the overall average in-hospital mortality in Italy therefore ranged around 30%.

Fear, scarce availability of PPE, 48-h waiting time for NPS and poor knowledge about the disease in the early days, possibly contributed to the virus spread, as did government uncertainty (81). The decision about who to intubate weighs on worldwide physician's jobs in catastrophic events: when patients exceed resources, physicians have to decide which patients will receive maximum benefits giving priority to those most likely to survive (82). In our experience, within days many ICU beds nevertheless became available also in neighboring areas, and resources sufficed.

Two positive points emerged. First, in a couple of weeks most research teams concentrated on Covid-19 with various approaches, leading to many research projects and studies. Last, preliminary treatment with immunoglobulin reduced mortality from 15 to 6% without inducing side effects, thus opening a possible important novel and inexpensive therapeutic option.

Overall, our experience describes Covid-19 pleomorphism at its onset. But what about the future? Possible late complications in patients who survive remain unknown. Death aside, they will be far reaching, at least in hospitalized patients. We expect that about 25% of patients might have prolonged and some persistent sequelae with impairment. These events will be challenging for physicians and for national health systems. So, we should be prepared to the impact of long-term sequelae of the disease and to the further Covid-19 waves.

## Data Availability Statement

The raw data supporting the conclusions of this article will be made available by the authors, without undue reservation.

## Ethics Statement

Ethical review and approval was not required for the study on human participants in accordance with the local legislation and institutional requirements. The patients/participants provided their written informed consent to participate in this study. Written informed consent was obtained from the individual(s) for the publication of any potentially identifiable images or data included in this article.

## Author Contributions

AP collected the material and drafted and edited the paper. All authors provided the clinical material and revised the final version of the manuscript.

## Conflict of Interest

The authors declare that the research was conducted in the absence of any commercial or financial relationships that could be construed as a potential conflict of interest. The reviewer SM declared a past co-authorship with one of the author AP to the handling editor at time of review.
